# The left-cradling bias and its relationship with empathy and depression

**DOI:** 10.1038/s41598-019-42539-6

**Published:** 2019-04-16

**Authors:** Gianluca Malatesta, Daniele Marzoli, Maria Rapino, Luca Tommasi

**Affiliations:** Department of Psychological, Health and Territorial Sciences, University “G. d’Annunzio” of Chieti-Pescara, Via dei Vestini, 31, I-66100 Chieti, Italy

## Abstract

Women usually cradle their infants to the left of their body midline. Research showed that the left cradling could be altered by affective symptoms in mothers, so that right cradling might be associated with a reduced ability to become emotionally involved with the infant. In this study, we assessed cradling-side bias (using family photo inspection and an imagination task), as well as depression and empathy, in 50 healthy mothers of 0–3 years old children. The main finding was that the strength of the left-cradling bias was negatively related with participants’ depression scores and slightly positively related with their empathy scores. Our results thus provide further evidence that cradling-side preferences can represent an evolutionary proxy of mother’s affective state, influencing the early development of the infant social brain and behaviour.

## Introduction

**Figure 1:**
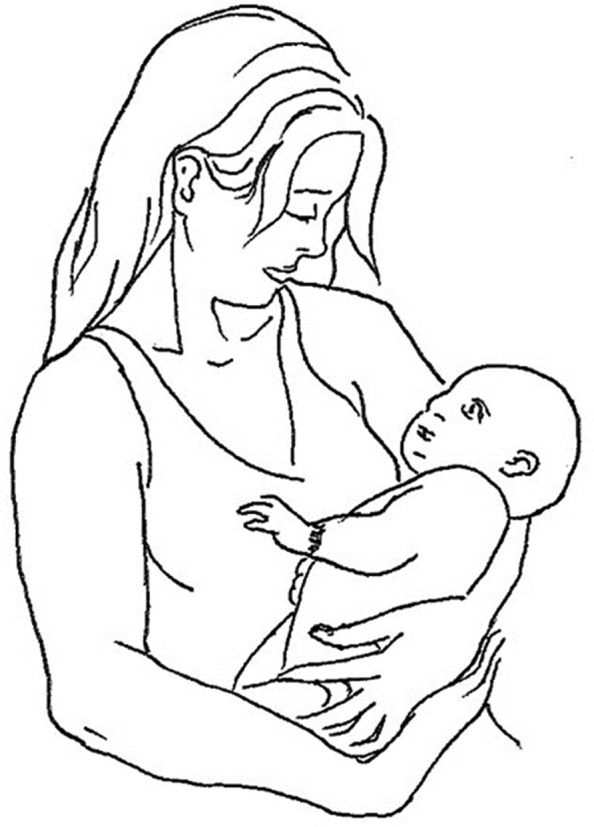
Graphic representation of left-cradling behaviour (courtesy of Rocco Cannarsa).

Maternal cradling is the female gender-specific motor behaviour wherein the mother holds an infant close to her body by using arms and hands (as shown in Fig. 1)^1^. Approximately 60–90% of women hold an infant to the left of the vertical midline of their own body, positioning the infant’s head in their left peripersonal hemispace, almost always bearing the weight with the left arm^2^. Interestingly, most research on cradling-side bias showed that such an asymmetry is independent of anatomical and postural variables, ethnic group and historical period^1–3^.

The occurrence of the left-cradling bias does not necessarily require the interaction with a living infant as the held “object”. Indeed, it was also consistently found in mothers, nulliparous females and children by using a life-like doll^4–7^, as well as in a task requiring to imagine holding an infant^8–13^. Additionally, Manning^14^, using an interesting methodology (suitable for answering to many experimental questions, as discussed below), examined many photographs from his colleagues’ family albums in which they were cradling their infants. Manning sorted the photographs according to the age of the cradled child and found that the strongest left-cradling bias (60–70%) was shown by females when holding children aged 0–3 months. In the other age groups (3–6 months, 6–12 months, 1–2 years, >2 years), females exhibited only a non-significant tendency to cradle on the left, the left-cradling bias appearing to decline after the third month of the infant’s life. Such findings are consistent with those of studies showing that the left-cradling bias in mothers is often stronger and more consistent during the first months after the infant’s birth. After this period, the left-cradling behaviour starts to decline and, according to some evidence, it could even be replaced by a right-cradling preference^15,16^.

Manning and Chamberlain^6^ proposed a neuropsychological explanation (“right-hemisphere hypothesis”) of the left-cradling bias according to which, during left-cradling interactions, the infant’s face is positioned on the left of the mother’s visual field so that visual information is mainly transmitted to the right brain hemisphere, which is involved in the perception and expression of emotion^17–19^. From the mother’s point of view, thus, the left cradling would facilitate the monitoring of her infant’s well-being or malaise cues through her left visual and auditory fields, which directly project to her right hemisphere, specialised for recognising emotional facial expressions^17,19^ such as crying^20^. From the infant’s point of view, the left cradling allows to get optimal emotional information through a constant access to the left side (i.e., the most expressive) of the mother’s face^18^. Interestingly, the existence of a population-level side bias in the positioning of offspring during parent-infant interactions can be observed in different species of apes^21^ and in several terrestrial and marine mammals^22^. Although the leftward or rightward direction of the bias in animals seems to be modulated by several factors such as the type of interaction (soothing, suckling or threatening), infant’s mobility (independent or dependent from the mother) and infant’s body orientation (towards the mother’s face or towards the environment), recent studies have shown that both mothers and infants seem to prefer a positioning fostering the communication of socio-emotional information through their right hemispheres^22–25^. Therefore, left cradling might be included in a repertoire of lateralised behaviours capable of improving individual biological fitness. The exact evolutionary pressures which shaped these behavioural asymmetries are still unclear, but animal studies seem to suggest a common pattern of lateralisation in vertebrates according to which the left hemisphere would be specialised for processing approach and manipulation responses, whereas the right hemisphere would be better specialised for avoidance responses, for detecting and reacting to threatening stimuli (such as predators), and for monitoring conspecifics (including infants; see Vallortigara & Rogers^26^ for a review).

In line with Giljov, Karenina and Malashichev^23^, it would be plausible to assume that the left-cradling behaviour should emerge (if not exclusively, at least mainly) when a face-to-face interaction between mother and child occurs. Indeed, this situation implies that most socio-emotional information is processed through the right hemisphere and a number of studies seem to corroborate the role of visual information in modulating the cradling-side preference. For example, Bourne and Todd^4^ (but see also Harris *et al*.^11^), in order to test the right-hemisphere hypothesis, asked right-handed males and females to cradle a life-like baby doll and to carry out a computerised version of the chimeric faces task. Their results indicated that left-cradling women, but not men, showed a significant right-hemispheric dominance for the perception of facial emotion, suggesting that brain asymmetry in emotional processing might indeed account for the left-cradling bias of right-handed females. Moreover, Huggenberger and collaborators^27^ found an interesting correspondence between the lateral preference for cradling in nulliparous females and the respective visual hemifield advantage for the perception of emotion in infant faces.

However, when children are held vertically over the mother’s shoulder (which is sometimes referred to as shoulder holding) rather than more horizontally on the mother’s arms (which is sometimes referred to as arm holding)^28^, one might expect that the occurrence of left cradling should be reduced because there is no visual interaction between mother and infant. On the other hand, it has been argued that vision is not the only sensory modality involved in cradling interactions. Indeed, there is some evidence that lateralised auditory perception is related to cradling-side preference^9,29^. Moreover, it has been suggested that the left-cradling bias could be due to asymmetric haptic sensitivity, the left side of the body being more sensitive than the right side^30^. In this regard, left cradling represents a kind of social touch aimed at facilitating the communication between mother and child, with the motor asymmetry and the early hemispheric specialisation for complex socio-affective behaviours acting synergistically^6,31^. Additionally, it should be noticed that humans show a strong population-level lateralisation (even though with a bias to the right rather than left side) in at least two other instances of social touch (i.e., embracing and kissing). Similarly to cradling, these lateralised behaviours are affected by several variables such as social pressures, handedness, and emotional context (see Ocklenburg *et al*.^32^ for a review), and a recent study proposed that the left-cradling preference could explain the fact that a left-turning bias is observed for parental kissing, but not for romantic kissing^33^.

Since the early observations reported by Salk^1^, and even before the growing amount of studies suggesting a potential link between the right-hemispheric dominance for emotion processing and the left-cradling bias, it was already clear that there might be an emotional component at the basis of the atypical reduction of the left-cradling preference observed in a minority of mothers^34^. The decrease in the left bias, indeed, seemed to be inversely related to psychological well-being. For example, mothers who were separated from their children for 24 hours did not show significant cradling-side preferences, and mothers who were separated from their children from 1 to 7 days showed a right-cradling bias^1^. Thus, interferences in early mother-infant interaction seem to cause a disruption of the usual leftward asymmetry. In line with this conclusion, right-cradling parents are more likely to have suffered from mental symptoms prior to child conception and right-cradling mothers are more likely to have been worried about parturition^35^. Moreover, mothers exhibiting a right-cradling bias appeared less committed in bodily contact with their infants^36^.

In a sample of mothers, Weatherill and colleagues^37^ also found a reduced left-cradling bias in those categorised as depressed according to the Beck Depression Inventory (BDI)^38^, as well as in those reporting domestic violence, and thus living in stressful conditions. On the basis of these results, the authors argued that, in depressed mothers, emotional signals from the infant might not cause a sufficient amount of arousal^39^, so that they do not tend to shift their attention to the left side, which in turn would result in a reduced left-cradling bias. Vauclair and Scola^40^ showed that the cradling-side bias was significantly related to the presence of affective symptoms (anxiety and depression) in mothers after delivery, right-cradling mothers reporting more affective symptoms than left-cradling ones. However, Reissland and colleagues^41^ provided evidence that stress, but not depression, could decrease the lateral asymmetry, and Donnot, Vauclair and Bréjard^42^ showed that the relationship between depression and right cradling is true only for bottle-feeding mothers, but not for breast-feeding ones. Finally, Morgan and colleagues^43^ failed to find a link between these variables. On the other hand, Scola and colleagues’^13^ longitudinal study confirmed that mother’s depression and stress as well as an older age of the infant may disrupt the left-cradling bias as assessed by using a real infant, affective symptom changes being predicted by changes in cradling-side preference two months after delivery. Moreover, similar results were observed for depression even when cradling was assessed by using a doll or an imagination task^44,45^.

Thus, the left-cradling bias might be closely associated to a higher quality of the caregiver-infant interaction and to a greater affective attunement between the cradling and the cradled individuals^34^. However, the typical pattern of cradling bias could be selectively altered by affective symptoms in mothers, so that right cradling might be associated with a reduced ability to become emotionally involved with the infant. As a matter of fact, the relationship between the right cerebral hemisphere and empathy^46–48^ is reasonably congruent with the role that the right hemisphere plays in interpreting facial emotional expressions, especially in female individuals^49^.

In this regard, Pileggi and collaborators^7^ hypothesised that the left-cradling bias might also be linked to abilities in empathic and socio-emotional processes, and recorded cradling preferences in individuals with autism spectrum disorders (ASD), whose deficits in social cognition, social attachment and empathy are particularly evident^50,51^. It was hypothesised that, if the left-cradling bias plays a positive role in emotional bonding and is typical of optimal and empathic relationships between the cradling and the cradled individuals, it would have been reduced or absent in ASD children. Indeed, such a lateral preference has also been observed — using a realistic doll — in typically-developing children since the age of about 4 years^5,52,53^. Pileggi and collaborators found that the control non-ASD children exhibited a significant left-cradling bias (82.5% of the sample), whereas the ASD participants did not exhibit any cradling-side preference (48% of the sample, a percentage not different from chance)^7^. Consistent findings were reported by Fleva and Kahn^54^, who tested adult participants by measuring their autistic-like traits on the basis of the “extreme male brain theory”^55^ and assessing their cradling bias with a life-like doll: a negative correlation between the left-cradling bias and the presence of autistic traits in typically developing adults was found.

The present study aimed to extend the results of different previous studies to a sample of mothers of young children. To this purpose, we made use of the Manning’s^14^ “family album” photographs method, whose reliability has never been assessed so far. Moreover, we tested whether the left-cradling bias of mothers is negatively and positively affected, respectively, by depression (as previously shown by many studies) and empathy (as suggested by the aforementioned studies involving ASD children or adults with autistic traits). Finally, we also used an imagination task for evaluating participants’ cradling-side bias by using, for the first time in the cradling literature, a “third-person” rather than a “first-person” imagination paradigm. Indeed, previous research successfully investigated the lateral preference by requiring the participant to imagine the cradling action as performed by him/herself  ^8–13^. In the present study, we assessed whether the motor representation of cradling behaviour is strong enough to emerge also when mothers are merely required to imagine another woman seen from behind (thus, from a back view) in the act of cradling. Compared with a front-view condition, a stronger correspondence between one’s own manual preference and the hand used by an imagined agent is observed during the imagination of others’ actions in a back-view condition, suggesting a larger involvement of motor representations in the latter case, which might be assimilated to a first-person perspective (although without any explicit request to resort to ones’ own motor habit), than in the former case, which might be assimilated to a third-person perspective^56^. Therefore, the imagination task aimed at testing (i) whether, when participants were asked to imagine a woman cradling an infant, the cradling side of the imagined woman would have matched the participants’ cradling-side preferences (as suggested by a series of studies)^56–58^, (ii) whether the cradling side of the imagined person would have been by itself a predictor of the participant’s depression or empathic tendencies, and (iii) whether the correspondence between the participants’ cradling-side preferences and the cradling side of the imagined woman would have been affected by empathic tendencies (as suggested by a previous study)^57^.

## Results

### Imagined cradling task

Participants imagined a significantly larger proportion of left-cradling rather than right-cradling women (32 [64%] left-cradling vs. 18 [36%] right-cradling; *χ*^2^_*(1)*_ = 3.92; *p* = 0.048).

### Photo cradling task

We collected data on 1570 photos (range per participant: 3–112; M = 31.4; SD = 22.18). Only participants who provided at least 4 maternal cradling photos (N = 49) were included in data analysis. A cradling laterality quotient (CLQ) was computed for each participant as *(right photos* − *left photos)*/*(right photos* + *left photos)* with participants scoring from −1 (all photos depicting left cradling) to +1 (all photos depicting right cradling) and 0 representing no cradling bias. The mean CLQ of the whole sample (M = −0.21; *t*_*(48)*_ = −3.333; *p* = 0.002) indicated a significant left-cradling bias (left-handers: M = −0.18; right-handers: M = −0.21). Participants scoring negatively on the CLQ were labelled as left-cradlers, and those scoring positively were labelled as right-cradlers. Depending on the CLQ score classification, a significantly greater proportion of participants were categorised as left-cradlers (32 [65.3%]) rather than right-cradlers (17 [34.7%]; *χ*^2^_*(1)*_ = 4.592; *p* = 0.032).

### Relationship between imagined cradling and CLQ score classification

A significantly larger proportion of participants imagined a cradling action corresponding (rather than non-corresponding) to their cradling-side preference as defined by the classification based on the CLQ from photos (36 [73.5%] vs. 13 [26.5%]; *χ*^2^_*(1)*_ = 10.796; *p* = 0.001). The analysis of participants’ CLQ classification from photos in relation to the imagined cradling task showed that left-cradling mothers imagined a left-cradling person (25 [78.1%]) more frequently than a right-cradling person (7 [21.9%]; *χ*^2^_*(1)*_ = 10.125; *p* = 0.001) and right-cradling mothers imagined a right-cradling person (11 [64.7%]) more frequently than a left-cradling person (6 [35.3%]; *χ*^2^_*(1)*_ = 1.471; *p* = 0.225), although in the latter case the difference was not significant, which might be due to the limited size of this subsample (indeed, the proportion of matches between one’s own cradling preference and the cradling side of the imagined action did not differ between left- and right-cradling mothers; *χ*^2^_*(1)*_ = 0.453; *p* = 0.501).

### Correlation between CLQ from photos and depression and empathy measures

**Figure 2:**
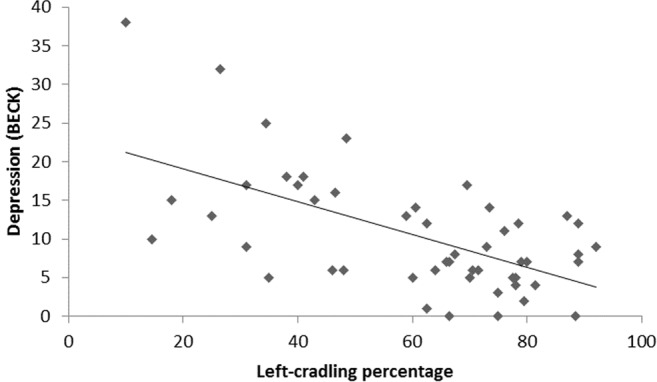
Scatterplot of depression scores and percentage of left cradling according to the CLQ from photos.

**Figure 3:**
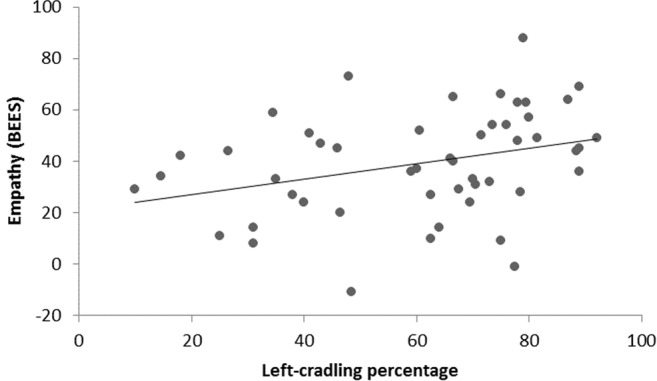
Scatterplot of empathy scores and percentage of left cradling according to the CLQ from photos.

Data analysis revealed a significant positive correlation between CLQ and depression scores as obtained by the administration of the Beck Depression Inventory II (BDI II^59^ *r*_*(49)*_ = 0.6; Bonferroni-corrected *p* = 0.00001; see Fig. 2) and a significant negative correlation between CLQ and empathy scores as obtained by the administration of the Balanced Emotional Empathy Scale (BEES^60^ *r*_*(49)*_ = −0.321; Bonferroni-corrected *p* = 0.049; see Fig. 3).

### Depression and empathic tendencies according to imagined cradling

Depression scores (BDI II) were higher in participants who imagined a right-cradling woman (M = 13.56) rather than a left-cradling woman (M = 8.69; *t*_*(48)*_ = 2.228; *p* = 0.031). As regards empathy scores (BEES), no significant difference was observed between participants who imagined a right-cradling (M = 34.05) or a left-cradling woman (M = 41.96; *t*_*(48)*_ = −1.333; *p* = 0.189).

### Empathic tendencies and correspondence between imagined cradling and CLQ score classification

No significant difference was observed between the empathy scores of participants whose cradling-side preferences (CLQ score classification) corresponded with those of the imagined woman (M = 38.94) and the empathy scores of participants whose cradling-side preferences did not correspond with those of the imagined woman (M = 37.61; *t*_*(47)*_ = 0.202; *p* = 0.84).

### Depression and empathic tendencies as a function of CLQ score classification and imagined cradling

When participants were split according to their cradling preference as assessed by the CLQ and the imagined cradling side, significant differences were observed in depression scores (*F*_*(3,45)*_ = 7.79; *p* = 0.0003). In particular, right-cradling mothers imagining a right-cradling woman showed higher levels of depression (M = 17) than left-cradling mothers imagining a left-cradling woman (M = 7; *p* = 0.0006) and left-cradling mothers imagining a right-cradling woman (M = 8.143; *p* = 0.044). Moreover, right-cradling mothers imagining a left-cradling woman showed higher levels of depression (M = 16) than left-cradling mothers imagining a left-cradling woman (M = 7; *p* = 0.024; Bonferroni correction was applied to adjust for multiple comparisons). Depression scores did not differ significantly between right-cradling mothers imagining a left-cradling woman and left-cradling mothers imagining a right-cradling woman (*p* = 0.213), but this is likely due to the very low sample size of these two groups. As regards empathic tendencies, no significant differences were observed when the same splitting procedure was applied (*F*_*(3,45)*_ = 0.95; *p* = 0.426).

## Discussion

In the present study, a left-cradling bias was observed for both the retrospective photo survey and the imagination task. Thus, our data corroborates the soundness of the retrospective photographic methodology in mothers, which indicates a left-cradling bias (65%) comparable to that usually reported in the literature, confirming the previous study by Manning^14^, who – to our knowledge – was the first and only author to successfully use such a method. Moreover, our study extends previous research using the imagination of one’s own actions^8–13^, showing that a left-cradling preference (64%) is also observed during the imagination of others’ actions, at least when the imagined person is visualised from the back. Therefore, the two measures of cradling bias adopted in the present study are consistent with one another and with the extant literature.

Our results also show a significant positive correlation between the strength of left-cradling preference and depression. Although this finding was rather predictable on the basis of previous literature^23,24,27^, this is the first time that such an association is revealed through the indirect photo inspection methodology. Therefore, our results seem to corroborate the idea that psychological distress contributes to the atypical reduction of the left-cradling bias shown by a minority of mothers^13,37^. More interestingly, a significant negative correlation between the strength of left-cradling preference and empathy was found. This is the first evidence of an association between mothers’ left-cradling bias and their ability to become emotionally involved with others (and, thus, with the cradled infant) by using a standardised instrument (i.e., the BEES)^60^, probably due to a right-hemispheric specialization in monitoring the infant’s well-being (e.g., refs^46,47,61^). This would be congruent with the role that the right hemisphere plays in the ability to interpret emotional expression from faces, which in turn seems to correlate with empathic abilities especially in females^49^. Neuroimaging and lesion studies also seem to strictly connect empathy with right-lateralised activity in the ventromedial prefrontal cortex^62^ and the parietal and temporal lobes^63^.

Therefore, in agreement with several empirical investigations linking lateral cradling preference with a right-hemispheric dominance for emotional processing (e.g., refs^4,6,27^), we hypothesise the existence of a relationship between the left-cradling bias and right-hemispheric social competencies such as empathy and attachment. Similar conclusions were drawn by Pileggi and colleagues^7^ and Fleva and Kahn^54^, who suggested that the left-cradling bias is linked to right-hemisphere-localised attachment processes (including empathy) allowing individuals to relate to others. Indeed, these authors observed that the left-cradling bias is absent in children with autism spectrum disorders and in adults with autistic traits (i.e., affected by milder impairments in socio-communicative and empathic relationships). Despite ASD aetiology is still unclear, such disorders have strong heritable and genetic underpinnings^64,65^. Interestingly, in a study on relatives, Manning and Denman revealed genetic influences on lateral cradling tendencies within families: women’s left cradling was found to be passed on to daughters^66^. Similarly, affective empathy and socio-emotional competencies seem to have heritable underpinnings^67–69^.

However, beside the role of genetics, the effects of experience should be taken into account. For example, remarkable results were found in a study shifting the focus from observed cradling to the cradling received during infanthood, which showed a reduced left bias for recognising faces in right-cradled individuals^70^. Moreover, Hendriks, van Rijswijk and Omtzigt^71^ asked mothers to cradle a doll with a camera fixed on its face, showing that the faces of right-cradlers were less visible from the “infant’s viewpoint” compared to those of left-cradlers both when they were looking up and when they were looking at the doll, which corroborates the notion that infant experience with cradling might affect the early development of cognitive abilities (such as face processing)^72^ crucial for social interactions. Actually, the results of past research^7,70^ suggest the importance of studies investigating whether any correlation exists between the cradling preference of the caregiver and the later emergence of ASD in the cradled individual, as already suggested by Jones^73^. According to this vision, left cradling might be considered as an early marker of the quality of the relationship and emotional closeness in the “cradling dyad”, and it would be plausible to hypothesise its negative correlation with the later diagnosis of ASD in infants.

Our results also show that the cradling side of the imagined women tended to match participants’ actual cradling preferences, as assessed by the CLQ from photos, consistent with previous findings on the imagination of actions^56–58^. This strongly suggests that participants actually put themselves in the imagined woman’s shoes during the imagined cradling task, an idea supported by the finding that reduced left-side preferences in the imagination task predicted participants’ depression scores. On the contrary, although we hypothesised a positive link between empathic abilities and the correspondence between imagined cradling side and participants’ cradling category according to the CLQ from photos on the basis of previous findings^57^, our data did not confirm our predictions that the proportion of matches and mismatches between actual and imagined cradling-side preferences should have been different for subjects with high and low self-reported empathy. However, the small size of this study might account for the inconsistency of results between the present and the previous study.

In summary, the present study confirmed that the left-cradling bias might be closely associated to the quality of caregiver-infant interaction and in particular to a greater emotional attunement between the cradling and the cradled individuals. On the contrary, right cradling might be associated with a reduced ability to become emotionally involved with the infant either because of the presence of depression symptoms or because of a lack of empathy (or because of both). Moreover, we could speculate that lateral cradling preferences in the mother might be a clue of her affective mental state and capacity to be involved in a positive emotional relationship with her child. Furthermore, it is likely that specific side biases experienced by the child during infanthood, such as in the cradling interaction, can set the developmental trajectory of socio-emotional attachment relationships in the child. Finally, our results assume particular importance in the light of recent findings indicating that received right cradling seems to alter the infant’s emotional processing in adulthood^70^, and further investigations are warranted in order to fully understand the importance of early lateral cradling experiences.

At this point, some caveats are necessary. First of all, it is important to remark that the CLQ from photos is not an index obtained from a direct observation, and thus it might be susceptible to many potential confounds that could intervene on the bias detection. In particular, photos can capture certain moments, but they are not necessarily fully descriptive of the actual cradling behaviour of mothers. A further limitation of the present study could be that, in order to keep the task the less invasive as possible to prevent participants from withdrawing, we did not require them to differentiate between the occurrence of horizontal or vertical holding. As highlighted in the introduction, the hypothesised effect of hemispheric asymmetries in emotional processing on the emergence of the left-cradling bias might be driven not only by the visual system but also by other sensory systems, and this could justify – at least in part – our decision to conflate shoulder and arm holding. However, we also point out how, since the beginning of cradling studies^1^, possible differences in the type of hold have been often neglected, probably because types of hold different from arm holding are rather uncommon^28,74,75^ (but see also ref.^40^). Furthermore, it should be remarked that studies investigating the relationship between cradling-side preferences and the same or similar variables as those examined in the present study either did not discriminate between or conflated the different types of hold, and nonetheless reported significant associations^7,13,37,41^. Analogous considerations can be made for the differentiation between functional (i.e., “cradling-while-doing-something-else” such as giving the infant a pacifier) and non-functional (cradling with the sole purpose of soothing/interacting with the infant) holding/cradling as related to handedness^76^, which again we did not consider for the sake of simplicity. Although many studies in left-handers showed reduced^9,77^ or typical^1,10,78^ – but not reversed – cradling, some clarifications are needed. Indeed, van der Meer and Husby^76^ tested a large sample of female and male participants of various ages, and showed that the lateralisation of the functional cradling of a doll was clearly affected by participants’ handedness. These authors concluded that right- and left-handed cradlers should exhibit, respectively, perfectly left and right biases during functional cradling activities. However, this account does not explain why most of the abovementioned authors failed to find a reversed (right) cradling bias in left-handed individuals. All that said, it is unlikely that these shortcomings might have determined the significant effects observed because any potential confounding factor would have reduced the association between lateral cradling preference and the variables of interest. Nonetheless, in future studies we plan to examine whether these findings can be modulated according to more specific subsets of motoric and postural interaction.

## Methods

### Participants

Fifty Italian mothers (age range: 24–44 years; M = 34.54; SD = 4.38; 2 left-handers) of children aged 0 to 3 years took part in the study. Prospective participants were contacted in person or by phone and asked whether they would be willing to participate in a study examining the mother-infant interaction. The experimenter met every mother individually in two separate sessions at a nursery or at their own homes. The second session took place about two weeks after the first. Neither invasive nor risky procedures were involved, and the data were analysed anonymously. Participants provided written informed consent at the beginning of the first session. The study was carried out in accordance with the principles of the Declaration of Helsinki and the guidelines of the AIP (Italian Association of Psychology) Ethical Code, and was approved by the Comitato Etico per la Ricerca “G. d’Annunzio”.

### Procedure and materials

Each participant followed the same procedure, as described below.

### Imagined cradling task

The experimenter, standing in front of the participant, made sure that she placed her hands palm-down on the knees or on a table, and that she did not cross her legs, arms or even fingers. Then the experimenter invited the participant to close her eyes and to imagine a female person seen from behind, without giving any further clue on the identity of the person to be imagined. Once the participant stated to “see” the person, the experimenter asked her to imagine that the woman was cradling an infant. Thereafter, when the participant stated to “see” the woman’s action clearly, the experimenter asked her to open her eyes and to imitate the imagined scene through the use of a life-like doll. Only one trial was administered to each participant. The trial took around 3–4 minutes to be accomplished.

### Questionnaires

In order to assess the participant’s hand preference, she was administered the Edinburgh Handedness Inventory^79^ (Italian version by Salmaso and Longoni)^80^, which measures laterality as a continuous variable ranging from −1 to +1 (from complete left-handedness to complete right-handedness, respectively). After completing the imagined cradling task, participants were asked to fill the following take-home tasks that did not require the experimenter’s presence.

Balanced Emotional Empathy Scale (BEES)^60^; Italian translation by Meneghini, Sartori and Cunico^81^. The BEES is a self-report 30-item questionnaire that assesses the tendency to share the emotional experiences of others and represents a measure of emotional empathy. Participants answer on a 7-point Likert scale with the scores calculated by summing the various items, and higher scores indicating higher empathic competencies.

Beck Depression Inventory II (BDI II)^59^; Italian translation by Ghisi and colleagues^82^. The BDI is a self-report 21-item questionnaire that assesses the severity of depression in adults and adolescents according to DSM-IV. Participants answer on a 4-point Likert scale with the scores calculated by summing the various items, and higher scores indicating more severe depressive symptoms.

### Photo cradling task

Participants were asked to consult their family photo albums, and specifically photographs in which they were cradling their children. Using the baby’s head as a reference point, they were required to make a note of the side (right or left) on which their child was held in each photo.

In the second session, participants returned the abovementioned surveys and a paper sheet indicating the number of photos in which they were depicted during right- and left-cradling interactions with their child. At the end of the session, participants were also debriefed about the study purpose by the experimenter.
